# Predicting Glass Transition Temperatures of Polyarylethersulphones Using QSPR Methods

**DOI:** 10.1371/journal.pone.0038424

**Published:** 2012-06-15

**Authors:** Ian Hamerton, Brendan J. Howlin, Grzegorz Kamyszek

**Affiliations:** Chemistry Department, Faculty of Health and Medical Sciences, University of Surrey, Guildford, Surrey, United Kingdom; NIH, United States of America

## Abstract

The technique of Quantitative Structure Property Relationships has been applied to the glass transition temperatures of polyarylethersulphones. A general equation is reported that calculates the glass transition temperatures with acceptable accuracy (correlation coefficients of between 90–67%, indicating an error of 10–30% with regard to experimentally determined values) for a series of 42 reported polyarylethersulphones. This method is quite simple in assumption and relies on a relatively small number of parameters associated with the structural unit of the polymer: the number of rotatable bonds, the dipole moment, the heat of formation, the HOMO eigenvalue, the molar mass and molar volume. For smaller subsets of the main group (based on families of derivatives containing different substituents) the model can be simplified further to an equation that uses the volume of the substituents as the principal variable.

## Introduction

Poly(arylene ether sulphone)s were originally developed during the 1960s following independent research work by the 3 M Corporation [1], Union Carbide [2] and the Plastics Division of ICI [3] to develop thermally stable thermoplastics suitable for engineering applications. The materials are highly aromatic polymers that comprise phenylene backbones bridged with heteroatoms (O, S) or groups (SO_2_, CH_2_, CH_3_CCH_3_, *etc.*), to offer thermal stability, good mechanical properties, creep resistance, and chemical resistance. These polymers have now reached a degree of maturity with many variants having been reported in both laboratory and commercial publications, and have been reviewed extensively [4]. Commercial products (*e.g.* Udel, Radel, and Victrex) are now available in a variety of grades to satisfy different high performance applications and are widely used. Poly(arylene ether sulphone)s display a wide range of glass transition temperatures (T_g_) influenced to a large degree by the chemical structure. Hence, polymers produced from dichlorodiphenylsulphone and simple bisphenols yield high T_g_ materials, typically in the range 180–230°C with the magnitude being influenced by the bulk of the substituents on the central carbon atom. The glass transition temperature is when the polymer goes from a glassy to a rubbery state. This is not a thermodynamic change of state so there is no exact value rather a range over which it occurs. Hence the experimentally determined value depends to a certain extent on how it is measured and quoted values can differ by plus or minus 10–20 K. There are a number of empirical equations to predict T_g_, the Fox equation, the Gordon and Taylor equation, the Kwei equation and first published in 2008, the equation of Brostow et al. [5], which uses a cubic polynomial based approach to predict the T_g_’s of polymer blends. The simulation of the thermal and mechanical properties of polymers is an area of growing interest. There are basically 2 main methods employed for this; the first of which is quantitative structure property relationships (QSPR) where group additive methods are used to derive values of the properties of interest. The second method is atomistic simulation which uses full atomic detail of the polymers. The prediction of thermal and mechanical properties in as yet unsynthesised polymers is beginning to be realised and we have been demonstrating this by the second method in a variety of thermosetting polymers such as epoxy resins [6], cyanate esters [7] and polybenzoxazines [8], as well as engineering thermoplastics [9], [10]. The QSPR method was initially pioneered by Van Krevelen culminating in a book published in 2009 [11]. In a previous publication [12], we reported the use of a quantitative structure property relationship (QSPR) to predict the Tg of a polymer of this type, but the model was severely limited by the size of the training set used to generate the QSPR equation. In the present work, a much more extensive study is reported extending the approach to an extensive range of poly(aryl ether sulphone)s.

## Methods

### The Data Set

A series of 66 polyarylene ether sulphones comprising between 1 and 4 phenylene rings in the structural repeat unit (SRU) were selected from a range of sources detailed in supporting information, Table S1. The rationale for selection was based on whether the structures offered a wide variety of different structural types and whether reliable, published empirical data were available for the polymer. The complete set of polyarylene ether sulphones and their corresponding T_g_ values **(**shown in order of increasing T_g_ **)** are given in supporting information, Table S1 [3], [5], [8], [10], [11], [13]–[47]. They are listed in terms of ID number. The ID numbers are used hereafter in the text.

**Table 1:** Variables in the equation for 57 SRUs.

Variables	Coefficent (B)	σ_B_
DF	−39.021	10.882
DM	−3.907	4.884
E_total_	−0.024	0.011
EIP_max_	−0.103	0.339
EIP_min_	0.165	0.259
ΔH_f_	0.059	0.117
HOMO	−16.454	16.574
LUMO	33.969	33.343
Mass	−0.042	0.143
V_chain_	0.964	0.558
constant	170.149	170.855

Multiple correlation coefficient R  = 0.688.

Coefficient of the multiple determination R^2^ = 0.474.

Adjusted R^2^ = 0.360.

Standard error  = 35.248.

**Table 2:** Variables in equation (2) for 42 SRUs, F  = 35.299.

Variables	Coefficient (B)	σ_B_
DF	−45.557	5.129
DM	2.844	2.137
E_total_	−0.029	0.005
ΔH_f_	0.099	0.043
HOMO	17.709	6.995
Mass	−0.176	0.064
V_chain_	1.301	0.272
constant	428.799	75.132

Multiple correlation coefficient R  = 0.932.

Coefficient of the multiple determination R^2^ = 0.869.

Adjusted R^2^ = 0.842.

Standard error  = 15.337.

**Table 3:** T_g_ values (°C) for different backbone motifs with the same number of bonds.

X	Motif 1 (3 ring sulphonewith × bridging group)	Motif 2(4 ring sulphonewith × bridging group)
-O-	180	145
-S-	180	146
-CH_2_-	180	146
>C = O	205	176
-SO_2_-	245	-

**Figure 1:**
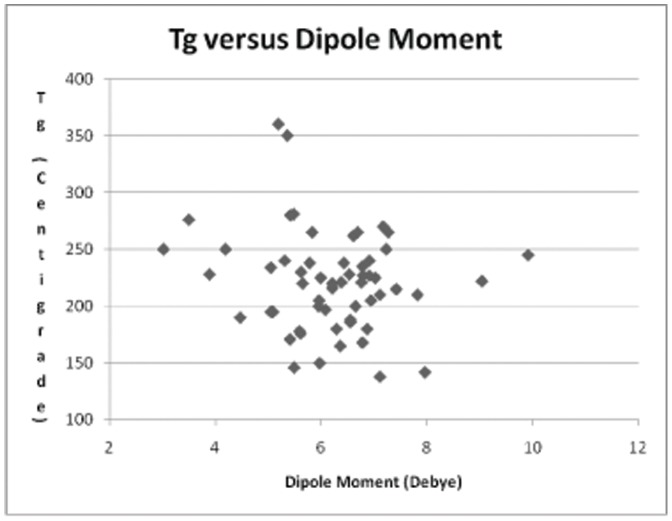
Scatter chart of dipole moment *versus* T_g_.

**Figure 2:**
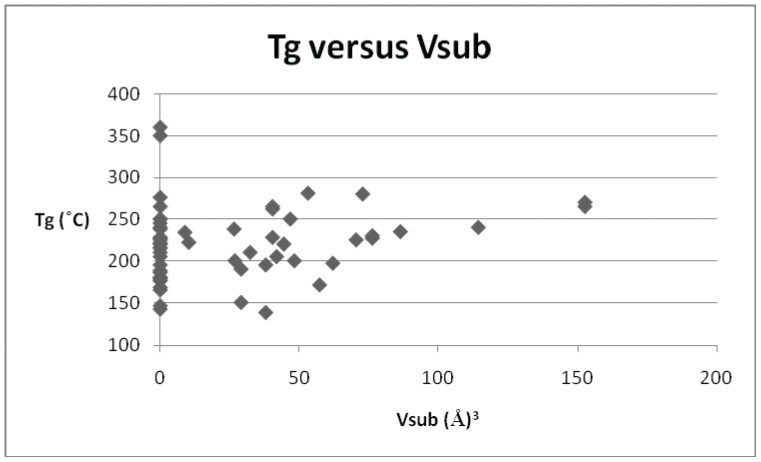
Scatter chart of T_g_ *versus* V_sub_.

**Figure 3:**
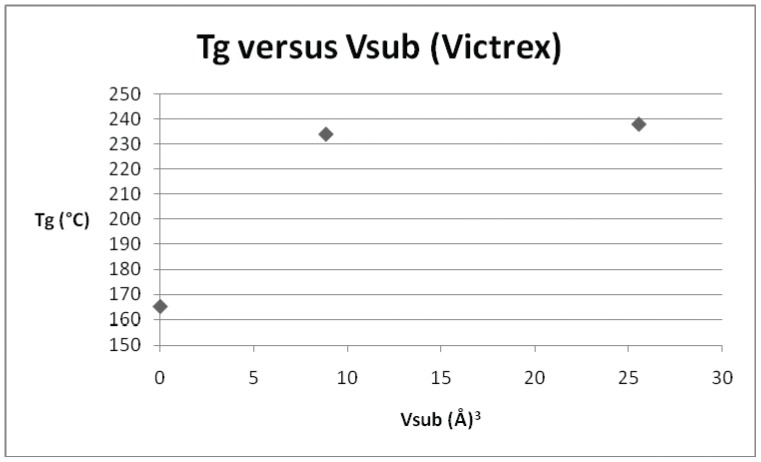
Plot of T_g_ *versus* V_sub_ for Victrex PES (repeat unit ID 5) and its derivatives.

**Figure 4:**
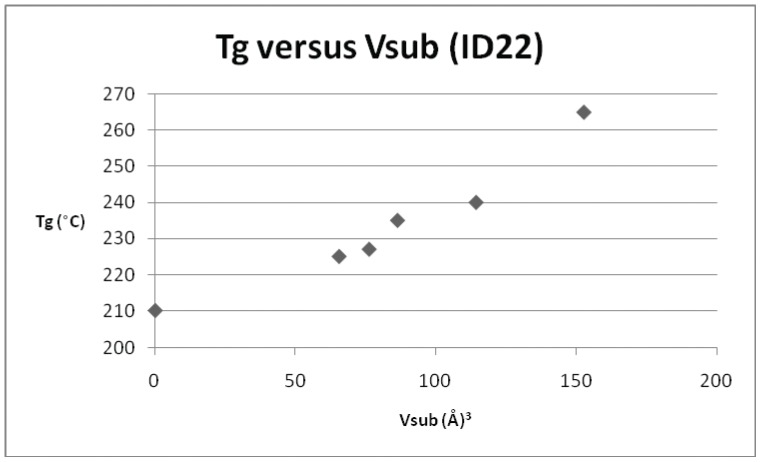
Plot of T_g_ *versus* V_sub_ for repeat unit ID 22 and its derivatives.

**Figure 5:**
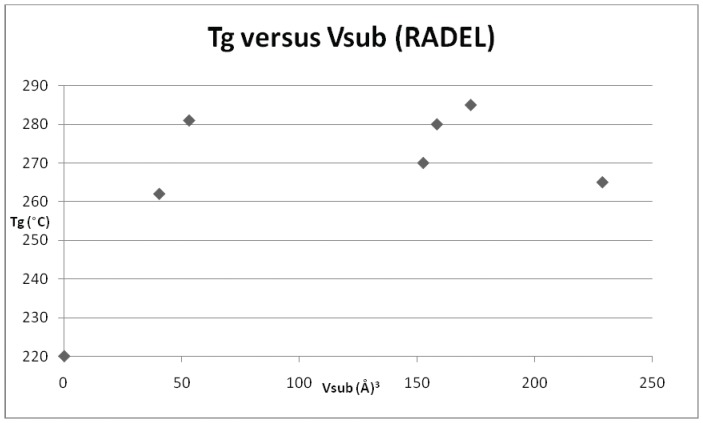
Plot of T_g_ *versus* V_sub_ for Radel™ R (repeat unit ID 26) and its derivatives.

**Figure 6:**
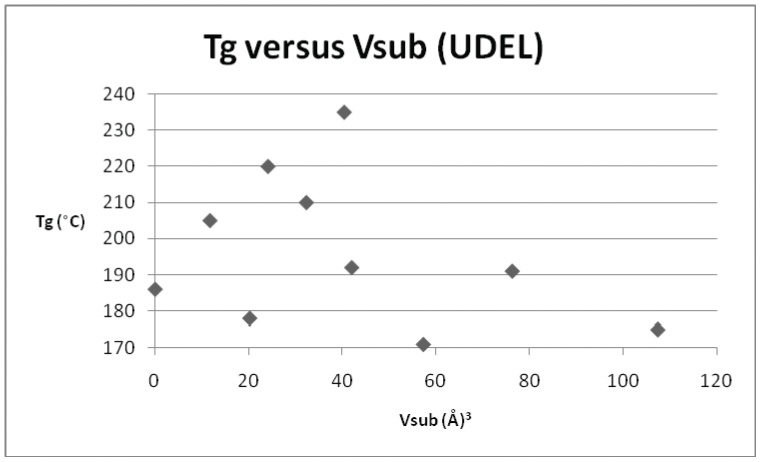
Plot of T_g_ *versus* V_sub_ for Udel™ polysulphone (repeat unit ID 12) and its derivatives.

**Figure 7:**
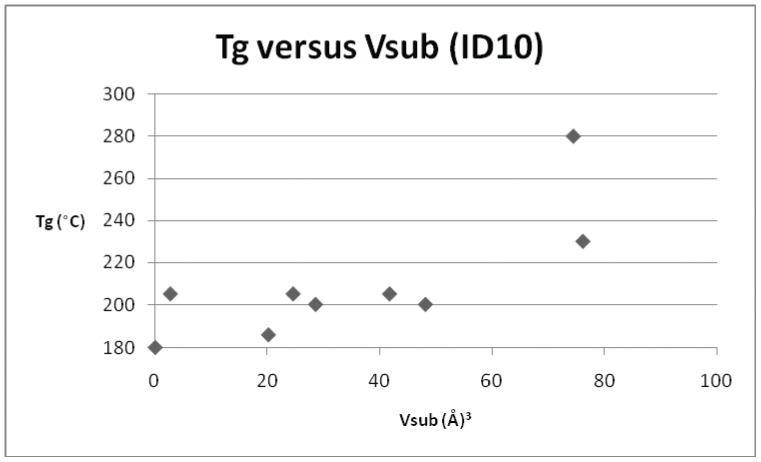
Plot of T_g_ *versus* V_sub_ for repeat unit ID 10 and its derivatives.

**Table 4:** T_g_ and V_sub_ values for the derivatives of the commercial polymer Victrex™ PES (shown below), repeat unit ID 5.

ID	T_g_ (°C)	V_sub_ (Å^3^)
5	165	0
38	234	8.86
40	238	25.56

**Table 5:** T_g_ and V_sub_ values for the derivatives of the repeat unit ID 22.

ID	T_g_ (°C)	V_sub_ (Å^3^)
22	210	0
31	225	65.5
34	227	76.2
39	235	86.3
43	240	114.3
51	265	152.5

**Table 6:** T_g_ and V_sub_ values for the derivatives of the commercial polymer Radel™ R, repeat unit ID 26.

ID	T_g_ (°C)	V_sub_ (Å^3^)
26	220	0
48	262	40.4
52	270	152.5
H	280	158.3
55	281	53.1
I	285	172.7
G	265	228.7

**Table 7:** T_g_ and V_sub_ values for the derivatives of the commercial polymer Udel™ polysulphone, repeat unit ID 12.

ID	T_g_ (°C)	V_sub_ (Å^3^)
12	186	0
D	205	11.7
1	138	37.9
C	192	42.0
23	210	32.3
9	178	20.2
27	220	24.1
7	171	57.3
F	235	40.4
G	175	107.3
B	191	76.2

**Table 8:** T_g_ and V_sub_ values for the derivatives of the repeat unit ID 10.

ID	T_g_ (°C)	V_sub_ (Å^3^)
10	180	0
12	186	20.2
19	200	48.2
18	200	28.6
21	205	2.7
E	205	24.6
20	205	41.8
37	230	76.2
54	280	74.5

**Table 9:** A and B coefficients and correlation coefficient (R) produced from equation (4).

ID	A	B	R
5	230.57	0.2905	0.982
22	204.99	0.3477	0.963
25	251.34	0.1345	0.548
12	-	-	0.091
10	181.24	0.2857	0.764

### Computational Details

The program Cerius^2^ (Accelrys, Inc.) using a Dell PC was employed to generate models of the SRUs detailed in supporting information, Table S1 using the amorphous builder module. All structures were fully minimised using conjugate gradients [48] until convergence was achieved. Electronic properties for the SRUs were calculated using MOPAC6 [49] with geometry optimisation, using RHF with the PM3 Hamiltonian. The results from the calculations have been summarised in supporting information, Table S2. The method was to perform multiple linear regression on all the data available to include the eigenvalues for the highest occupied molecular orbitals (HOMOs), lowest unoccupied molecular orbitals (LUMOs), global energy minima, dipole moments, and electrostatic isopotentials (EIPs); the latter were calculated with the QUANTA program [50]. The parameters were chosen carefully on the basis of knowledge of the factors that generally affect the T_g_ of the polymers. The global energy minimum represents the overall stability of the polymer, and it seems reasonable to conclude that a more stable polymer would have a higher value for its T_g_. This is reflected in the negative coefficient for this parameter, as the more negative the energy minimum the more stable the polymer. The EIPs represent the ability of a polymer to form hydrogen bonds. The presence of hydrogen bonds is likely to add stability and hence the larger the magnitude of EIP, the greater the T_g_. A similar trend can be seen with the dipole moments. Larger energy differences between the HOMO and LUMO indicate a more stable polymer. Hence, a more negative value for the HOMO and a more positive value for the LUMO would both contribute to a higher value of T_g_. The statistical calculations were performed within the SPSS program [51].

## Results and Discussion

### Development of Quantitative Structure Property Relationships

The method used was based on QSPR theory, in which an attempt is made to describe the activity or reactivity within a set of compounds by means of a mathematical formalism that incorporates structure-dependent parameters. In QSPR it is assumed that the effects of the various parameters included are additive, and that they vary in a linear manner. Multiple linear regression (MLR) can be performed on the data to obtain an equation relating the property under investigation (in this case the glass transition, T_g_) to the parameters from the set of test data. In this study, the method used was based on the work of Hopfinger and Koehler [52], who assumed that bulk properties of polymers can be described as a sum of the properties (*e.g.* HOMO and LUMO eigenvalues, global energy minimum, *etc.*) of the repeat unit. Thus, only the SRU needs to be modelled (1).
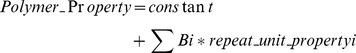
(1)where B_i_  =  coefficient in the equation and i  =  the name of the SRU.

A series of selected SRU properties were determined to represent the parameters that might influence the magnitude of T_g_:

heat of formation (ΔHf),
dipole moment (DM),
total energy (E_total_),
HOMO eigenvalue (HOMO),
LUMO eigenvalue (LUMO),
electrostatic isopotential maximum (EIP_max_),
electrostatic isopotential minimum (EIP_min_),
molar mass of SRU (Mass),
sum of the cubes of the van der Waals’ radii of all atoms in the SRU [53] (proportional to volume) (V_tot_),
sum of the cubes of the van der Waals’ radii of atoms in the substituents of the SRU (V_sub_),
sum of the cubes of the van der Waals’ radii of atoms in the backbone of the SRU (V_chain_  =  V_tot_-V_sub_),
degree of freedom: number of bonds in the backbone of the SRU around which any rotation is possible (DF).

In all regressions vchain only was used instead of vtot and vsub because absolute values of the B coefficients of these parameters were very similar. The first seven properties represent electronic and polar properties associated with the SRU, and the remainder describe its volume, mass and relative flexibility. The complete set of calculated parameters for each SRU (along with the reported empirical Tg) is given in supporting information, Table S2. Initially a multiple regression was performed on all the parameters (supporting information, Table S2) for the entire data set (of 57 SRUs) and yielded the following regression data (Table 1):

The magnitude of the various coefficients reflects the importance of each parameter in the regression equation. It can clearly be seen that this is not a robust regression as the standard deviations of the coefficients are in some cases greater than the coefficients themselves. This is further reflected in the poor value of the correlation coefficient which shows that these parameters only model 68% of the data and the high value of the proportionality constant which is large at 170 and acts to provide most of the variation in T_g_. From these data, it was apparent that not all of the parameters in the regression were making a significant contribution. Indeed in the case of four variables (*DF*, *E_total_*, *EIP_min_*, and *V_chain_*) a correlation coefficient of R  = 0.671 was obtained (only 0.017 less than for all the variables). At the same time, the value of F for this regression was 10.62, which was only three times greater than F calculated for 4 degrees of freedom, for the 52 SRUs at the 0.01 probability level, which was 3.7 (at the 0.99 confidence limit). This assumes that all of the variables are independent which may not be the case with e.g. *E_total_* and *EIP_min_* but nevertheless provides some confidence in the reliability of the regression to yield equation (2):
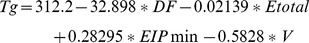
(2)


Therefore the *DM*, *EIP_max_*, *ΔH_f_*, *HOMO*, *LUMO* and *Mass* parameters were all removed before a second multiple regression was performed for the entire data set (of 57 SRUs) to yield the following regression data:

Multiple correlation coefficient R  = 0.671
Coefficient of the multiple determination R_2_ = 0.450
Adjusted R2 = 0.407
Standard error  = 33.906

After rejecting 15 SRUs, ID numbers 1, 4, 5, 6, 7, 13, 24, 25, 29, 33, 47, 50, 53, 54 and 56 from the original data set (on the basis that their predicted T_g_ values deviated by more than 10% from the empirical data), the multiple regression was repeated using the same seven parameters but for a set of 42 SRUs. This second regression yielded the following statistics (Table 2):

The marked improvement in the correlation coefficient (from 67% to 93.2%) indicates that the T_g_ is well replicated using the following equation, although not all the factors are taken into account as 7% of the data are still unaccounted for.
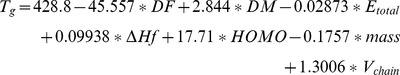
(3)


The small standard deviations of most of the coefficients and the value of F at 32.9, compared to the tabulated value of F at the 0.01 probability level for 7 degrees of freedom and 34 SRUs at 3.3 indicate that the equation is reasonably well determined with a probability greater than 99.5%. Addition of the remaining variables gave only very small improvements in the correlation coefficient. The smallest deviations are for the T_g_ values in the range 190–220°C, presumably due to the population being greatest in this temperature range.

The greatest problems with the dataset were associated with estimating the flexibility of the chain, the parameter representing the degrees of freedom and the volume of the chain. The methods used were quite simplistic and insufficient to tackle the wider range of SRUs. Consequently, a smaller set of compounds was derived from the full dataset in order to find a correlation between the flexibility and T_g_ (Table 3) and between the volumes of substituents and T_g_. Five base repeat units were identified (SRU ID 5, 10, 12, 22, 26) and their derivatives with various substituents were examined in more detail. The introduction of a stiffening group into the backbone is known to raise T_g_ and the data in Table 3 demonstrate how T_g_ is enhanced by the incorporation of more rigid bridges having lower rotational freedom or the potential for dipole-dipole interactions between adjacent polymer chains. An attempt was made to discern any relationships between the various calculated parameters and the magnitude of T_g_. Figure 1 shows the scatter chart for the values of T_g_ *versus* dipole moment for the smaller subset of sulphone derivatives identified above. It is clear that no discernible relationship was evident, and this was the case for all of the parameters, save the volume of the substituent (V_sub_), which is displayed in Figure 2. In this case, although there is some scatter evident at lower values of substituent volume, there does appear to be a trend of increasing T_g_ with increasing volume. Polyarylethersulphones in common with many synthetic polymers contain areas of amorphous chains and areas of crystallinity, the relative proportion of these regions can differ with the material and its method of preparation. This in turn would affect the glass transition values determined for the materials. Hence there is expected to be a scatter in the degree of correlation of the materials chosen and we have chosen to concentrate on those that give the best correlations. On this basis a series of graphs (Figures 3, 4, 5, 6, 7) were plotted for V_sub_ *versus* T_g_ for each of the individual derivatives. In addition to the original dataset, a series of 9 poly(arylether sulphone)s (originally published in reference 10), were also incorporated into the later plots and regressions. Three examples of polysulphones (based on the commercial polymer Victrex™) were included in the original data set and the parameters are presented in Table 4. Figure 3 depicts the relationship for Victrex™ PES (repeat unit 5) from which it can be seen that there is a linear correlation for T_g_ with increasing V_sub_, albeit with a very small data set (n = 3), which is to be expected for only 3 examples. The SRU ID 22 is better represented in the data set and six examples are included in Table 5. Figure 4 depicts a clear and strong linear relationship for T_g_ with increasing V_sub_, emphasising the utility of this approach. Unfortunately, although there is more information (with 7 data points) for the commercial polymer Radel™ R (ID 26) and its derivatives (Table 6), the plot of T_g_ and V_sub_ for (Figure 5), provides a less convincing relationship. There is an increasing trend discerned, but with large scatter in the data. The third commercial polymer (Udel™, ID 12) has the largest single data set (comprising 11 datapoints) (Table 7). This data set is the most disappointing and least convincing: the plot of T_g_ and V_sub_ for (Figure 6) shows no discernible relationship.

Finally, and in contrast, repeat unit ID 10 (once again well represented in the data set with 9 examples, Table 8) is well modelled with an increasing trend in T_g_ with V_sub_ shown in Figure 7. Having established that the parameter V_sub_ offers a reasonably good guide for the magnitude of T_g_ for the majority of the poly(arylene ether sulphone)s, the regressions were run again for repeat unit using the greatly simplified equation:
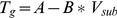
(4)


The coefficients are given in Table 9 and these demonstrate the strength of the correlations for the top two SRUs, where a correlation greater than 90% is obtained, but this clearly only works for relatively simple structures. This indicates that T_g_ can be correlated with the volumes of the substituents using a very simple equation but only for a small set of repeat units, excluding for example biphenylene or methylene bridges in the SRU. In this paper an equation (derived from multiple linear regression) is presented relating the glass transition temperatures of poly(aryl ether sulphone)s to various atomistic parameters. Several molecular modelling techniques were used for building and minimization of the structures and subsequent molecular orbital calculations on these structures. The equation is useful in providing molecular insight into the observed T_g_ values of poly(aryl ether sulphone)s (*i.e.* the equation points to the importance of chain stiffness and substituent volume in determining the T_g_).

## Supporting Information

Table S1 Poly(arylene ether sulphone)s examined in this work (shown in order of increasing T_g_).
(DOC)

Table S2 The complete set of parameters from the molecular orbital calculations for each SRU (for ID refer to Table S1).
(DOC)

## References

[1] Vogel HA (1963). Polyarylsulphone Polymers.. British Patent: 1,060,546.

[2] Farnham AG, Johnson RN (1973). Polyarylsulphone Polymers.. British Patent: 1,078,234.

[3] Jones MEB (1962). Manufacture of Polysulphones.. British Patent: 1,016,245.

[4] Cotter RJ (1995). Engineering Plastics. A Handbook of Polyarylethers, Basel: Gordon and Breach Publishers.. 357 p.

[5] Brostow W, Chiu R, Kalogeras IM, Vassilikou-Dova A (2008). Prediction of Glass transition Temperatures: Binary Blends and Copolymers.. Materials Letters.

[6] Howlin BJ, Hall SA, Hamerton I, Billaud C, Baidak A (2012). Improvement of molecular simulation techniques to predict properties of crosslinked epoxy resins.. PLOS1: in press.

[7] Howlin BJ, Hamerton I, Klewpatinond P, Shortley H, Takeda S (2006). Developing predictive models for polycyanurates through a comparative study of molecular simulation and empirical thermo-mechanical data.. Polymer.

[8] Hamerton I, Howlin BJ, Mitchell AL, Hall SA, McNamara LT, Ishida H, Agag T (2011). Using molecular simulation to predict the physical and mechanical properties of polybenzoxazines..

[9] Hamerton I, Heald CR, Howlin BJ (1996). Molecular simulation of the comparative flexibility of bridging linkages in poly(aryl ether sulfone)s and poly(aryl ether ketone)s from a study of isolated oligomers.. Die Macromol Chemie Theor Simul.

[10] Hamerton I, Heald CR, Howlin BJ (1996). Molecular modelling of a Polyarylethersulfone under bulk conditions.. Modell Simul Mater Sci Eng.

[11] Van Krevelen DW, Nijenhuis TK (2009). Properties of Polymers. Their Correlation with Chemical Structure, Their Numerical Estimation and Prediction from Additive Group Contribution. Amsterdam: Elsevier.. 1000 p.

[12] Hamerton I, Howlin BJ, Larwood V (1995). Development of Quantitative Structure-Property Relationships for Poly(Arylene Ether)s.. J Mol Graphics.

[13] Ueda M, Toyota H, Ochi T, Sugiyama J, Yonetak K (1993). Synthesis and Characterization of Aromatic poly(ether sulfone)s containing pendant sodium-sulfonate groups.. J Polym Sci A.

[14] Ueda M, Ito T (1991). Synthesis of Aromatic Poly(ether sulfone)s by Nickel-catalyzed Coupling Polymerization of Aromatic Dichlorides.. Polym J.

[15] Podkoscielny W, Dethloff J, Dethloff M, Brunn J (1991). Aliphatic-aromatic Polyethersulfones.2. Polycondensation Products of bis-(4-hydroxyphenyl)-sulfone and bis(4-chloromethylphenyl) compounds - Synthesis and Structure.. Angew Makromol Chemie.

[16] Attwood TE, Barr DA; King T, Newton AB, Rose JB (1977). (Poly(arylene ether sulfones) by Polyetherification.2. Polycondensations.. Polymer.

[17] Vogel HA (1970). Polyarylsulfones, Synthesis and Properties.. J Polym Sci A-1.

[18] Hsaio BS, Gardner KH, Matheson RR (1991). Structure, crystallisation and melting of poly(aryl ether ketone ketone) (PEKK). Part II: Crystallisation and melting.. ACS Polym Prep.

[19] Robeson LM, Farnham AG, McGrath JE (1975). Polym.. Prep.

[20] Johnson RN, Farnham AG, Clendinning RA, Hale WF, Merriam,CN (1967). Poly(aryl ethers) by Nucleophilic Aromatic Substitution.1. Synthesis and Properties. J Polym Sci.A-1 5: 2375.. DOI:10.1002/pol.1967.150050.

[21] Robeson LM, Crisafull ST (1983). Microcavity Formation in Engineering Polymers Exposed to Hot Water.. J Applied Polym Sci.

[22] O’Shea FX, Cornell RJ (1974). Aromatic polyether-polythioether-polysulfone thermoplastics.. Can Patent 946,091.

[23] Ghosal K, Chern RT (1992). Aryl-nitration of Poly(phenylene oxide) and Polysulfone - Structural Characterization and Gas-permeability.. J Membr Sci.

[24] Kamps KMP, Teunis HA, Wessling M, Smolders CA (1992). Gas-transport and Sub-t(g) Relaxations in Unmodified and Nitrated Polyarylethersulfones.. J Membr Sci.

[25] Lee JA, Hogen-Esch TE (1993). Polym Prep.

[26] McHattie JS, Koros WJ, Paul DR (1991). Gas-transport Properties of Polysulfones.1. Role of Symmetry of Methyl-group Placement on Bisphenol rings.. Polymer.

[27] Goh SH, Lau WW, Lee CS (1992). Miscibility of Poly(4-vinyl pyridine) with Polysulfone and Carboxylated Polysulfone.. Polym Bull.

[28] Schmidt M, Maurer FHJ (1998). Pressure–volume–temperature properties and free volume parameters of PEO/PMMA blends.. J Polym Sci B Polym Phys.

[29] Attwood TE, Dawson PC, Freeman.JL, Hoy LRJ, Rose JB (1981). Synthesis and Properties of Polyaryletherketones.. Polymer.

[30] Clendinning RA, El-Hibri MJ, Matzner M, Kwiatkowski GT (1988). Polym Prep.

[31] Rose JB (1974). Preparation and Properties of Poly(arylene ether sulfones).. Polymer.

[32] Matsuo S, Murakami T, Takasawa R (1993). Synthesis and Properties of New Crystalline Poly(arylene ether nitriles).J Polym Sci A Polym Chem.

[33] Mohanty DK, Hedrick JL, Gobetz K, Johnson BC, Yilgor I (1982). Poly(arylene ether sulfones) and related materials via a potassium carbonate, n-methyl pyrrolidone process.. Polym Prep.

[34] Cotter RJ (1989). Novel Poly (arylethers).US Patent 4,849,503..

[35] Baron L, Blank DR (1970). Synthesis and Properties of Some Aromatic Polythioethers.. Makromol Chem.

[36] Kim WJ, Hay AS (1992). Soluble Poly(ether ketone)s and Poly(ether sulfone)s From Phenyl-substituted Hydroquinones.. J Macromolecular Science Pure Appl Chem.

[37] Hartmann LA (1979). High Molecular Weight Polyethersulfones.. US Patent 4,156,068.

[38] Tam CM, Dal-Cin M, Guiver MD (1993). Polysulfone Membranes.4. Performance Evaluation of Radel-a/pvp Membranes.. J Membr Sci.

[39] Attwood TE, Barr DA, King T, Newton AB, Rose JB (1977). Poly(arylene ether sulfones) by Polyetherification.2. Polycondensations.. Polymer.

[40] Rose JB (1968). Poly(arylene sulphones) and Poly(arylene ketones).. Chem Ind.

[41] Robeson M, Farnham AG, McGrath JE (1975). Effect of Structure on Dynamic Mechanical-Behavior of Poly(aryl ethers).. Abstr Papers ACS.

[42] Maresca LM, Chao HS (1984). Poly (arylether)s.. US Patent number 4,473,684.

[43] Wham GK, Hay AS (1991). Soluble homopoly(aryl ether ketone) (HPAE) from 2,2′,3,3′,6,6′-hexaphenyl-4,4′-diphenol (HPDP) and 4,4′-difluorobenzophenone (DFK). Polym.. Prep.

[44] Andrews SM (1992). Synthesis, characterization, and blends of high temperature poly(arylether sulfone)s.. J Polym Sci Polym Chem.

[45] Percec V, Wang JH, Oishi Y, Feiring AE (1991). Synthesis of Aromatic Polyethers by Scholl Reaction.4. Homopolymerization and Copolymerization of Alpha, Omega-bis [4-(1-naphthoxy) phenylsulfonyl]perfluoroalkanes.. J Polym Sci, Polym Chem.

[46] Rose JB (1974). Synthetic Routes to Polyethersulphones.. Chimia.

[47] Ivin KJ, Rose JB (1968). in Adv Macromol Chem W Pasika (Ed.).

[48] Fletcher R, Reeves CM (1964). Function Minimization by Conjugate Gradients.. J Comput.

[49] Stewart JJP (1990). Special Issue - MOPAC - A Semiempirical Molecular-Orbital Program.. J Comput-Aided Mol Des.

[50] (2012). QUANTA, Accelrys Inc.. , accessed.

[51] (2012). http://www-01.ibm.com/software/analytics/spss/.

[52] Koehler MG, Hopfinger AJ (1989). Molecular Modeling of Polymers.5. Inclusion of Intermolecular Energetics in Estimating Glass and Crystal-melt Transition-Temperatures.. Polymer.

[53] Pauling L (1960). The Nature of the Chemical Bond, Cornel University Press: Ithaca, 664 p..

