# Supplementary figures and images for: Predicting Glass Transition Temperatures of Polyarylethersulphones Using QSPR Methods
Source: PLoS One. 2012 Jun 15;7(6):e38424. doi: 10.1371/journal.pone.0038424 (PMC3376152; doi:10.1371/journal.pone.0038424)

| ID number | Structural Repeat Unit | Tg (°C) | Reference |
| --- | --- | --- | --- |
| 1 |  | 138 | [13] |
| 2 |  | 142 | [14] |
| 3 |  | 146 | [15] |
| 4 |  | 150 | [11] |
| 5 |  | 165 | [16] |
| 6 |  | 168 | [17] |
| 7 |  | 171 | [18] |
| 8 |  | 176 | [13] |
| 9 | 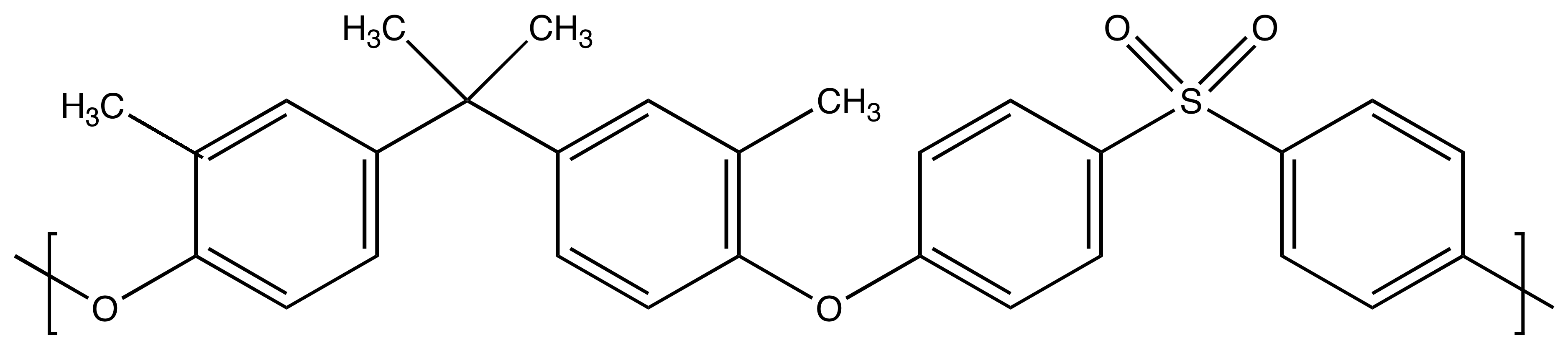 | 178 | [19] |
| 10 |  | 180 | [20] |
| 11 |  | 180 | [8] |
| 12 |  | 186 | [21] |
| 13 |  | 188 | [22] |
| 14 |  | 190 | [23] |
| 15 |  | 195 | [24] |
| 16 |  | 195 | [25] |
| 17 |  | 197 | [26] |
| 18 |  | 200 | [8] |
| 19 |  | 200 | [8] |
| 20 |  | 205 | [8] |
| 21 |  | 205 | [8] |
| 22 |  | 210 | [8] |
| 23 |  | 210 | [27] |
| 24 |  | 215 | [28] |
| 25 |  | 216 | [29] |
| 26 |  | 220 | [30] |
| 27 |  | 220 | [15] |
| 28 |  | 221 | [31] |
| 29 |  | 221 | [32] |
| 30 |  | 222 | [33] |
| 31 |  | 225 | [34] |
| 32 |  | 225 | [35] |
| 33 |  | 227 | [36] |
| 34 |  | 227 | [16] |
| 35 |  | 228 | [37] |
| 36 |  | 228 | [13] |
| 37 |  | 230 | [8] |
| 38 |  | 234 | [38] |
| 39 |  | 235 | [24] |
| 40 |  | 238 | [3] |
| 41 |  | 238 | [35] |
| 42 |  | 240 | [39] |
| 43 |  | 240 | [24] |
| 44 |  | 245 | [8] |
| 45 |  | 250 | [40] |
| 46 |  | 250 | [5] |
| 47 |  | 250 | [8] |
| 48 |  | 262 | [41] |
| 49 |  | 265 | [19] |
| 50 |  | 265 | [42] |
| 51 |  | 265 | [24] |
| 52 |  | 270 | [43] |
| 53 |  | 276 | [44] |
| 54 |  | 280 | [26] |
| 55 |  | 281 | [45] |
| 56 |  | 350 | [46] |
| 57 |  | 360 | [47] |
| A |  | 175 | [10] |
| B |  | 191 | [10] |
| C |  | 192 | [10] |
| D |  | 205 | [10] |
| E |  | 205 | [10] |
| F |  | 235 | [10] |
| G |  | 265 | [10] |
| H |  | 280 | [10] |
| I |  | 285 | [10] |

Supplement: Table S1 — Poly(arylene ether sulphone)s examined in this work (shown in order of increasing Tg). (DOC) [file pone.0038424.s001.doc]
