# Supplementary material for: Predicting Glass Transition Temperatures of Polyarylethersulphones Using QSPR Methods
Source: PLoS One. 2012 Jun 15;7(6):e38424. doi: 10.1371/journal.pone.0038424 (PMC3376152; doi:10.1371/journal.pone.0038424)
Supplement: Table S2 — The complete set of parameters from the molecular orbital calculations for each SRU (for ID refer to Table S1). (DOC) [file pone.0038424.s002.doc]

**Table 2. The complete set of parameters from the molecular orbital calculations for each SRU (for ID refer to supporting information, table 1)**

Key

T_g_ glass transition temperature (°C)

DF degree of freedom - number of rotatable bonds in structural repeat unit

ΔH_f_ Heat of formation of structural repeat unit (kJ/mol)

DM dipole moment (Debyes)

E_Total_ total energy of structural repeat unit (kJ/mol)

HOMO Eigenvalue for highest occupied molecular orbital (eV)

EIP_max_ maximum electrostatic surface potential

EIP_min_ minimum electrostatic surface potential

Mass mass of structural repeat unit (g)

V_total_ sum of van der Waals radii of all atoms in structural repeat unit (nm^3^)

V_sub_ sum of van der Waals radii of all atoms in substituent, if present, (nm^3^)

V_chain_ sum of van der Waals radii of all atoms in backbone of structural repeat unit (nm^3^)

| ID | T_g_ (°C) | DF | ΔH_f_ (kJ/mol) | DM(D) | E_Total_ (kJ/mol) | HOMO (eV) | LUMO (eV) | EIP_max_ | EIP_min_ | Mass (g) | V_total_ (nm^3^) | V_sub_ (nm^3^) | V_chain_ (nm^3^) |
| --- | --- | --- | --- | --- | --- | --- | --- | --- | --- | --- | --- | --- | --- |
| 1 | 138 | 7 | -202 | 7.11 | -26733 | -9.17 | -1.69 | -132.90 | -336.50 | 532 | 20.22 | 3.79 | 16.43 |
| 2 | 142 | 7 | -122 | 7.96 | -4927 | -9.31 | -0.57 | -72.33 | -201.00 | 400 | 16.29 | 0 | 16.29 |
| 3 | 146 | 10 | -24 | 5.49 | -19387 | -9.29 | -0.48 | -39.67 | -176.10 | 426 | 18.80 | 0 | 18.80 |
| 4 | 150 | 6 | -18 | 5.97 | -17195 | -9.21 | -0.73 | -75.24 | -241.60 | 347 | 14.90 | 2.91 | 12.00 |
| 5 | 165 | 4 | -208 | 6.36 | -11040 | -9.59 | -0.58 | -63.51 | -181.30 | 232 | 8.73 | 0 | 8.73 |
| 6 | 168 | 6 | -66 | 6.78 | -17492 | -9.15 | -0.81 | -50.36 | -177.40 | 384 | 16.01 | 0 | 16.01 |
| 7 | 171 | 8 | -329 | 5.41 | -22906 | -9.09 | -0.41 | -70.89 | -194.10 | 514 | 22.33 | 5.73 | 16.61 |
| 8 | 176 | 9 | -3.5 | 5.61 | -18744 | -9.11 | -0.46 | -37.11 | -175.90 | 412 | 18.0 | 0 | 18.0 |
| 9 | 178 | 8 | -235 | 5.59 | -21846 | -9.02 | -0.49 | -70.27 | -191.70 | 470 | 20.50 | 0 | 17.12 |
| 10 | 180 | 8 | -145 | 6.29 | -19346 | -9.15 | -0.53 | -87.00 | -203.10 | 414 | 17.12 | 0 | 17.12 |
| 11 | 180 | 8 | -96 | 6.87 | -19946 | -9.15 | -0.49 | -87.00 | -203.10 | 416 | 16.60 | 0 | 16.60 |
| 12 | 186 | 8 | -169 | 6.55 | -20595 | -9.15 | -0.52 | -73.52 | -186.90 | 442 | 18.80 | 0 | 16.80 |
| 13 | 188 | 6 | -20 | 6.55 | -15043 | -9.06 | -0.57 | -49.45 | -175.90 | 340 | 13.10 | 0 | 13.10 |
| 14 | 190 | 8 | -214 | 4.47 | -23653 | -9.81 | -1.29 | -94.17 | -252.20 | 487 | 19.60. | 2.91 | 16.60 |
| 15 | 195 | 8 | -235 | 5.09 | -26709 | -9.94 | -1.37 | -120.90 | -323.90 | 532 | 20.22 | 3.80 | 16.43 |
| 16 | 195 | 6 | -186 | 5.05 | -16483 | -10.35 | -0.94 | -75.85 | -197.30 | 348 | 13.63 | 0 | 13.63 |
| 17 | 197 | 8 | -244 | 6.08 | -23591 | -9.04 | -0.48 | -60.42 | -191.20 | 510 | 22.64 | 6.24 | 16.43 |
| 18 | 200 | 8 | -198 | 6.65 | -21220 | -9.15 | -0.54 | -63.69 | -191.00 | 456 | 19.50 | 2.68 | 16.80 |
| 19 | 200 | 8 | -9 | 5.95 | -23196 | -9.17 | -0.54 | -58.56 | -191.20 | 504 | 21.60. | 4.82 | 16.78 |
| 20 | 205 | 8 | -174 | 5.96 | -22339 | -9.14 | -0.53 | -60.74 | -192.40 | 482 | 21.00 | 4.20 | 16.80 |
| 21 | 205 | 8 | -252 | 6.94 | -20443 | -9.52 | -0.73 | -84.03 | -204.60 | 428 | 17.10 | 0 | 17.10 |
| 22 | 210 | 6 | -227 | 7.11 | -15493 | -9.48 | -0.51 | -75.52 | -200.10 | 374 | 12.65 | 0 | 12.65 |
| 23 | 210 | 8 | -501 | 7.82 | -23456 | -9.09 | -0.94 | -101.90 | -300.70 | 486 | 19.84 | 3.23 | 16.61 |
| 24 | 215 | 6 | -155 | 7.42 | -17602 | -8.97 | -0.82 | -73.34 | -198.60 | 374 | 15.00 | 0 | 15.00 |
| 25 | 216 | 5 | -115 | 6.21 | -14268 | -9.35 | -0.94 | -42.96 | -176.00 | 308 | 12.37 | 0 | 12.37 |
| 26 | 220 | 7 | -125 | 5.65 | -18721 | -8.99 | -0.59 | -72.61 | -198.20 | 400 | 16.30 | 0 | 16.30 |
| 27 | 220 | 8 | -840 | 6.21 | -26497 | -9.16 | -0.90 | -126.30 | -350.50 | 530 | 20.90 | 4.45 | 16.43 |
| 28 | 221 | 4 | -195 | 6.38 | -11040 | -9.68 | -0.49 | -70.18 | -196.12 | 232 | 8.73 | 0 | 8.73 |
| 29 | 221 | 6 | -94 | 6.76 | -17602 | -8.94 | -0.83 | -72.09 | -199.80 | 374 | 15.00 | 0 | 15.00 |
| 30 | 222 | 6 | -46 | 9.04 | -16597 | -9.82 | -0.92 | -78.70 | -189.90 | 393 | 13.30 | 1.03 | 12.28 |
| 31 | 225 | 6 | -375 | 7.02 | -20493 | -9.23 | -0.48 | -75.40 | -203.50 | 388 | 19.51 | 7.04 | 12.48 |
| 32 | 225 | 8 | -129 | 5.99 | -19052 | -8.86 | -0.77 | -40.55 | -168.50 | 448 | 17.28 | 0 | 17.28 |
| 33 | 227 | 6 | -154 | 6.79 | -17602 | -8.92 | -0.80 | -74.64 | -199.70 | 374 | 14.96 | 0 | 14.96 |
| 34 | 227 | 6 | 2 | 6.91 | -21947 | -9.39 | -0.47 | -76.39 | -192.30 | 476 | 20.10 | 7.62 | 12.48 |
| 35 | 228 | 6 | -184 | 3.89 | -16483 | -10.13 | -1.13 | -89.06 | -196.10 | 348 | 13.63 | 0 | 13.63 |
| 36 | 228 | 8 | -280 | 6.53 | -21848 | -8.99 | -0.45 | -75.82 | -191.80 | 470 | 20.90 | 4.04 | 16.80 |
| 37 | 230 | 8 | -155 | 5.62 | -25797 | -9.12 | -0.56 | -58.49 | -191.00 | 566 | 24.10 | 7.62 | 16.50 |
| 38 | 234 | 4 | -259 | 5.05 | -14098 | -10.39 | -1.51 | -98.52 | -259.70 | 277 | 9.44 | 0.90 | 8.60 |
| 39 | 235 | 6 | 124 | 6.78 | -22572 | -9.28 | -0.44 | -77.50 | -191.70 | 490 | 20.80 | 8.63 | 12.13 |
| 40 | 238 | 4 | 103 | 5.78 | -13075 | -9.15 | -1.06 | -61.34 | -188.50 | 282 | 11.05 | 2.70 | 8.40 |
| 41 | 238 | 6 | -146 | 6.43 | -17602 | -8.76 | -0.87 | -74.01 | -199.30 | 374 | 15.00 | 0 | 15.00 |
| 42 | 240 | 4 | -5 | 5.31 | -10590 | -9.24 | -0.82 | -137.50 | -152.70 | 280 | 9.10 | 0 | 9.10 |
| 43 | 240 | 6 | 126 | 6.91 | -25174 | -9.37 | -0.45 | -69.63 | -191.70 | 552 | 23.60 | 11.44 | 12.13 |
| 44 | 245 | 8 | 414 | 9.91 | -21934 | -9.17 | -0.78 | -95.86 | -216.10 | 464 | 17.50 | 0 | 17.50 |
| 45 | 250 | 4 | -235 | 4.19 | -11042 | -9.19 | -0.78 | -71.86 | -170.60 | 232 | 8.73 | 0 | 8.73 |
| 46 | 250 | 6 | -265 | 3.02 | -19606 | -9.58 | -1.22 | -83.76 | -228.00 | 422 | 15.90 | 0 | 15.90 |
| 47 | 250 | 8 | -105 | 7.23 | -22833 | -9.15 | -0.52 | -59.04 | -191.20 | 494 | 21.45 | 4.68 | 16.80 |
| 48 | 262 | 7 | -261 | 6.61 | -21223 | -8.66 | -0.65 | -74.84 | -198.90 | 456 | 19.63 | 4.04 | 15.60 |
| 49 | 265 | 5 | -110 | 6.69 | -14628 | -9.19 | -0.74 | -57.55 | -177.40 | 308 | 12.40 | 0 | 12.40 |
| 50 | 265 | 8 | -478 | 5.83 | -22452 | -9.66 | -0.85 | -99.41 | -225.40 | 520 | 20.82 | 4.04 | 16.80 |
| 51 | 265 | 6 | 241 | 7.27 | -28401 | -9.31 | -0.44 | -67.70 | -191.80 | 628 | 27.20 | 15.25 | 11.96 |
| 52 | 270 | 7 | 327 | 7.17 | -31629 | -8.89 | -0.62 | 57.53 | -187.70 | 705 | 30.85 | 15.25 | 15.60 |
| 53 | 276 | 7 | -233 | 3.50 | -20727 | -9.89 | -1.12 | -77.31 | -213.80 | 48 | 17.20 | 0 | 17.20 |
| 54 | 280 | 8 | 154 | 5.42 | -25668 | -8.99 | -0.58 | -56.42 | -192.10 | 564 | 24.05 | 7.30 | 16.78 |
| 55 | 281 | 7 | 821 | 5.48 | -22937 | -8.72 | -0.79 | -68.58 | -188.10 | 500 | 20.91 | 5.31 | 15.60 |
| 56 | 350 | 2 | -153 | 5.36 | -6586 | -10.31 | -0.59 | -45.51 | -153.30 | 140 | 4.82 | 0 | 4.82 |
| 57 | 360 | 2 | -145 | 5.19 | -10909 | -9.42 | -0.87 | -63.77 | -187.70 | 198 | 8.38 | 0 | 8.38 |
